# Nanosheet composed of gold nanoparticle/graphene/epoxy resin based on ultrasonic fabrication for flexible dopamine biosensor using surface-enhanced Raman spectroscopy

**DOI:** 10.1186/s40580-020-00225-8

**Published:** 2020-05-05

**Authors:** Mahmoud A. Hussein, Waleed A. El-Said, Bahaa M. Abu-Zied, Jeong-Woo Choi

**Affiliations:** 1 Chemistry Department, Faculty of Science, King Abdulaziz University, P.O. Box 80203, Jeddah, 21589 Saudi Arabia; 2 Chemistry Department, Faculty of Science, Assiut University, Assiut, 71516 Egypt; 3 College of Science, Department of Chemistry, University of Jeddah, P.O. Box 80327, Jeddah, 21589 Saudi Arabia; 4 Center of Excellence for Advanced Materials Research (CEAMR), King Abdulaziz University, P.O. Box 80203, Jeddah, 21589 Saudi Arabia; 5 Department of Chemical and Biomolecular Engineering, Sogang University, 35 Baekbeom-Ro, Mapo-Gu, Seoul, 04107 Republic of Korea

**Keywords:** Epoxy resin, Graphene nano-sheets, Dopamine biosensor, Neurotransmitters, Gold nanoparticles/graphene/epoxy, Surface-enhanced Raman scattering

## Abstract

Construction of a fast, easy and sensitive neurotransmitters-based sensor could provide a promising way for the diagnosis of neurological diseases, leading to the discovery of more effective treatment methods. The current work is directed to develop for the first time a flexible Surface-Enhanced Raman Spectroscopy (SERS) based neurotransmitters sensor by using the ultrasonic-assisted fabrication of a new set of epoxy resin (EPR) nanocomposites based on graphene nanosheets (GNS) using the casting technique. The perspicuous epoxy resin was reinforced by the variable loading of GNS giving the general formula GNS/EPR_1–5_. The designed products have been fabricated in situ while the perspicuous epoxy resin was formed. The expected nanocomposites have been fabricated using 3%, 5%, 10%, 15% and 20% GNS loading was applied for such fabrication process. The chemical, physical and morphological properties of the prepared nanocomposites were investigated by using Fourier transforms infrared spectroscopy, X-ray diffraction, Thermogravimetric analysis, Differential Thermal gravimetry, and field emission scanning electron microscopy methods. The GNS/EPR_1–5_ nanocomposites were decorated with a layer of gold nanoparticles (Au NPs/GNS/EPR) to create surface-enhanced Raman scattering hot points. The wettability of the Au NPs/GNS/EPR was investigated in comparison with the different nanocomposites and the bare epoxy. Au NPs/GNS/EPR was used as a SERS-active surface for detecting different concentrations of dopamine with a limit of detection of 3.3 µM. Our sensor showed the capability to detect low concentrations of dopamine either in a buffer system or in human serum as a real sample.

## Introduction

Dopamine (DA) is one of the most important catecholamine neurotransmitters that have a vital role in the transmission of nerve impulses. Several physiological processes and illnesses including Parkinsonism, Schizophrenia, and Huntington’s disease [1] are related to the changes in the DA levels. Therefore, observing the concentrations of DA receive great attention. Several electrochemical and optical biosensors have reported for the detection of DA [1–3]. The rigid conventional sensors were disadvantaged because of their rigidity from capturing analytes and their deformation by thinned [4, 5]. On the other hand, flexible sensors could capture target analytes more efficiently and showed higher quality signals. Several flexible electrochemical sensor platforms have reported for either in vitro or in vivo monitoring of different biomarkers and neurotransmitters [6–13]. Recently, some researches focused on the fabrication of flexible SERS for their biological applications, which including in situ detection based on wrapped the flexible sensor on a solid substrate; besides, few studies have reported the uses of flexible sensors for the direct detection of analytes in the liquid phase [14].

Significant interest has been noticed in the past few decades for polymer composite materials which, are related to organic–inorganic hybrid components. This is fundamentally attributed to their expected and unexpected final properties, which merge the basic characteristics of each component in one new fabricated material [15–17]. To understand what is happening in such unification of polymers from variable groups with inorganic nanofillers we have to believe the appearance of synergistic effects, which drive the researchers to produce innovative multifunctional new materials [18]. Polymer composite materials are the shape of high-performance products that be produced by an easy method. The broad zone of polymer composite materials applications and its considerable behavior have been implicated considerable awareness in the past few decades. Polymer composite materials have been also frequently distinguished and display fundamental properties due to its low coast and numerous ameliorations in its complete performance [19–25]. They should also expand other demands, for example, better mechanical performance, high operating temperature range, electrostatic discharge, and sufficient chemical resistance through others [26]. Furthermore, graphene nanosheets (GNS), carbon nanotubes, and other carbon-based nanomaterials are widely utilized with a variety of polymers in different forms due to its enormous properties in different fields of application. Amazing exceptional properties that will create new materials with excellent properties such as high specific surface areas, unique size distributions. Such properties permit graphene and/or CNTs to be used in different industrial fields of applications such as sensing, catalysts, solar cells, composites, medical applications, photonics, and fuel cells. Moreover, GNS has electrical conductivity properties sufficient to change completely the conducting behaviors of any materials [27–35].

Raman spectroscopy technique represents a nondestructive analytical technique that has high selectivity, quick response in addition to its ability to provide rich information about the target species without needing sample preparation. The weak Raman intensity has restricted its applications for detecting trace species. Several techniques were applied to enhance the Raman signals; among these techniques, surface-enhanced Raman scattering (SERS) is the most common one [36]. Uses of the SERS technique enable the detection of many important targets at very low concentration levels with high selectivity and sensitivity in the presence of metal nanoparticles [37]. Several noble metals including silver (Ag), gold (Au) or copper (Cu) nanostructures or their composites with different sizes and shapes have been used as high active-SERS agents [38–42]. The chemical nature, size, shape, and spacing of the metal nanomaterials have the main influences on the intensity of the SERS signals. Ag nanostructured showed the highest SERS signals, but the low stability of Ag nanostructures hindering its SERS applications. Although there is notable progress in SERS research, many challenges achieving a repeatable and quantitative SERS signal and fabrication of uniform and stable nanoparticles hindering its development. Using of colloidal solutions of the noble NPs results in a non-uniform enhancement of the Raman signals due to the accumulations of the NPs. Thus, numerous substrates modified with noble NPs were used as SERS agents. In the current work, we have used the ultrasonic-assisted technique for the fabrication of a new set of epoxy resin (EPR) nanocomposites with different amounts of graphene nanosheets (GNS) including 3%, 5%, 10%, 15% and 20% of GNS using the casting technique. The perspicuous epoxy resin was reinforced by the variable loading of GNS giving the general formula GNS/EPR_1–5_. Then we have used these composites sheets as flexible substrates for developing SERS substrates based on decorated these GNS/EPR_1–5_ sheets with Au NPs. We investigate the use of surface-enhanced Raman spectroscopy (SERS) based sensors for the rapid detection of dopamine neurotransmitters. According to our knowledge, it is the first time to develop a flexible SERS sensor of detecting dopamine neurotransmitters.

## Experimental

### Materials and chemicals

Commercially obtained Epikote-1001 × − 75% (2642) epoxy together with crayamid—100% (2580) epoxy hardener was applied as pure epoxy resin; they were also used as obtained without additional purification. Epikote:crayamid (1:1) weight by weight was adjusted as the exact mixing ratio for pure epoxy processing and fabrication. Spectroscopic grade chloroform was obtained from Sigma-Aldrich and used without any additional purification. Furthermore, GNS, dopamine and chloroauric acid tetrahydrate (HAuCl_4_·4H_2_O) were also purchased from Sigma-Aldrich and were also used as received. Any other chemicals or materials used were also obtained from a known source, besides, were used as received.

### Preparation of pure epoxy resin film

A thin film of pure epoxy resin was easily fabricated by ultrasonic assistance as well as casting technique as reported in our previous work [32, 43, 44]. The following steps were applied: In 50 mL beaker, a fixed weight of 1 g of Epikote-1001 was dissolved in 25 mL of chloroform. Besides that, 1 g of crayamid hardener was also dissolved in similar mL of chloroform in another beaker. Both solutions were exposed to ultrasonicator for 10 min before mixing in a closed container. The whole mixture was constantly exposed to ultrasonicator for an additional 10 min while its top is closed. This sonicated mixture was poured carefully into a Petri dish and left overnight at room temperature for solvent evaporation. The pure epoxy thin film was collected easily and dried in the oven at 40 °C.

### Fabrication of GNS/EPR_1–5_ nanocomposites

A new set of GNS/EPR nanocomposites with a general formula GNS/EPR_1–5_was simply fabricated using casting technique and ultrasonic assistance as well. The proposed products were fabricated in situ while the perspicuous epoxy resin was introduced. 3%, 5%, 10%, 15% and 20% loading of GNS was applied for such fabrication process with respect to the total weight of the perspicuous epoxy resin. In a typical procedure, GNS/EPR_1–5_was fabricated as follows: In three different beakers variable weights of GNS, 1 g of Epikote-1001 and 1 g of crayamid hardener were separately dissolved in 20 mL of chloroform for each. Such solutions were exposed to ultrasonicator for 10 min before mixing in a closed container. The total mixture was continuously exposed to ultrasonicator for an additional 10 min while its top is closed. This sonicated mixture was poured carefully into a Petri dish and left overnight at room temperature for solvent evaporation. A thin film of GNS/EPR_1–5_ was collected easily each time and dried in the oven at around 40–50 °C. These procedures were repeated five times with a different weight of GNS was introduced each time. The designed compositions for GNS/EPR_1–5_ formulations were given in Table 1.

**Table 1:** Designed compositions and symbols for NEAT EPR and its GNS/EPR_1–5_ graphene-containing nanocomposites

Sample	EPR (weight, g)	G loading %, (weight, g)
Pure EPR	(2 g)	–
GNS/EPR_1_	(1.96)	3%, (0.06)
GNS/EPR_2_	(1.90)	5%, (0.10)
GNS/EPR_3_	(1.80)	10%, (0.20)
GNS/EPR_4_	(1.70)	15%, (0.30)
GNS/EPR_5_	(1.60)	20%, (0.40)

### Fabrication of Au NPs/GNS/EPR_1–5_ nanocomposites modified sheet as a dopamine-based biosensor

Au NPs/GNS/EPR sheet was prepared based on a chemical reduction method in which the GNS/EPR sheets were immersed solution of 1 mM of HAuCl_4_ and then added few drops of 0.1 M of cooled NaBH_4_ as a reducing agent. Then rinse the substrate with DIW and dried. To develop the dopamine sensor, fifty microliters of the dopamine solution into the modified substrate and kept for 6 h at 4 °C and then reins the substrate with DIW.

### Characterization and identification techniques

Powder X-ray diffractograms were determined in the *2θ* range from 5 to 80° with the aid of Philips diffractometer (type PW 103/00) using the Ni-filtered CuKα radiation. FT-IR spectra were examined by using ATR smart part technique within the wavenumber range from 4000 to 400 cm^−1^ using the Thermo-Nicolet-6700 FT-IR spectrophotometer. Thermal analysis, the TGA curve was recorded with a TA instrument apparatus model TGA-Q500 using a heating rate of 10 °C min^−1^ under nitrogen atmosphere over the temperature range of 21–700 °C. The average masses of the samples were 5–10 mg. The morphological features were characterized by field emission scanning electron microscope (JEOL JSM-7600F, Japan). The FE-SEM samples were prepared by evaporating a dilute solution of each nanocomposite on a smooth surface of the aluminum foil, and subsequently coating it with gold–palladium alloy. The microscope was operated at an accelerating voltage of 5 kV and a 4 mm work distance carbon film.

The chemical composition of the prepared resins and their different composites, as well as the SERS efficiency of the Au NPs/GNS/EPR_1–5_, were studied by Raman spectroscopy using a Bruker Senterra Raman microscope (Bruker Optics Inc., Germany) with 785 nm excitation, 1200 rulings mm-1 holographic grating, and a charge-coupled device (CCD) detector. The accumulation time was 3 s with a power of 50 mW. Five scans of 5 s from 200 to 2000 cm^−1^ were measured and the mean of these scans was used.

## Results and discussion

### Synthesis and characterizations of different GNS/EPR_1–5_ nanocomposites sheets

Variable characterization techniques are utilized to recognize the chemical structure and to confirm the formation of these expected products.

**Fig. 1:**
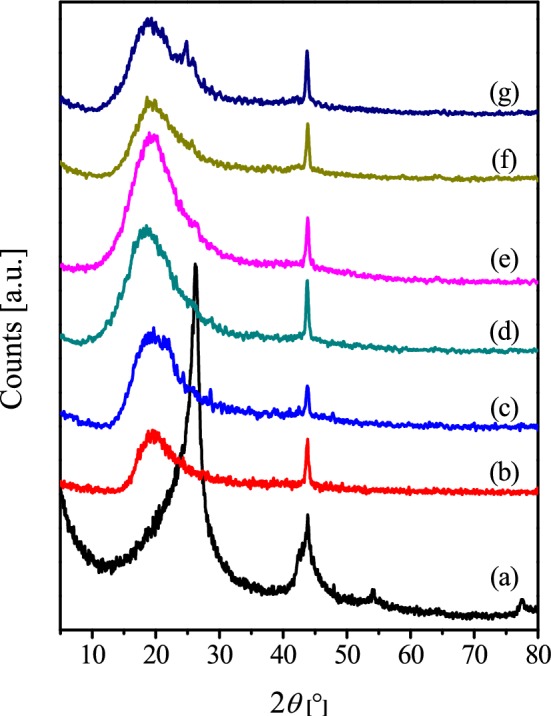
XRD patterns of GNS (**a**), neat EPR (**b**), GNS/EPR_1_ (**c**), GNS/EPR_2_ (**d**), GNS/EPR_3_ (**e**), GNS/EPR_4_ (**f**) and GNS/EPR_5_ (**g**)

To investigate the structures of the prepared GNS/EPR_1–5_ nanocomposites and the dispersion of GNS in their matrix, XRD analysis has been performed. Figure 1 shows the XRD patterns for GNS, neat epoxy and their prepared nanocomposites with various GNS contents. The diffractogram of the as-received neat GNS (Fig. 1a) shows four broad-diffraction peaks at 2*θ *= 26.20°, 43.80°, 54.33°, and 77.45°, which correspond to the interlayer spacing of 0.3398, 0.2065 and 0.1687 nm. These reflections match well with those reported for GNS [45, 46]. The diffractogram of neat EPR (Fig. 1b) shows a broad reflection at 2*θ *= 13–32° and a sharp one at 2*θ *= 43.85°. The obtained diffractograms for the composites with GNS content of 3 and 5 wt% resemble very closely to the XRD pattern of the neat epoxy (Fig. 1c, d). In this context, Zaman et al., [47] demonstrated that epoxy-graphene composites contain low graphene loading (~ 0.5 wt%) exhibit sharp XRD peak at 26.5° attributable to layered crystalline GnPs, which indicates the persistence of the graphene-layered structure. Epoxy/reduced graphene oxide (RGO) and ternary epoxy/RGO/powdered rubber (PR) composites showed the absence of such diffraction peak and the presence of wide one at 2*θ *= 5°–28°, due to the scattering of the cured epoxy molecules, which indicates amorphous nature of these composites [48]. Two points could be highlighted from the absence of GNS diffraction peaks for our nanocomposites with loading 3–5%: (i) the reflections of G for the low GNS-content composites could be masked by the resin signal, and (ii) this indicates the homogeneous intercalation of epoxy chains into the GNS interlayer together with the exfoliation of the graphene sheets in the epoxy matrix. A similar argument was proposed by Wan et al. [49, 50] for their epoxy composites filled with graphene oxide (GO), reduced graphene oxide (RGO) and diglycidyl ether of bisphenol-A functionalized GO (DGEBA–f–GO).

**Fig. 2:**
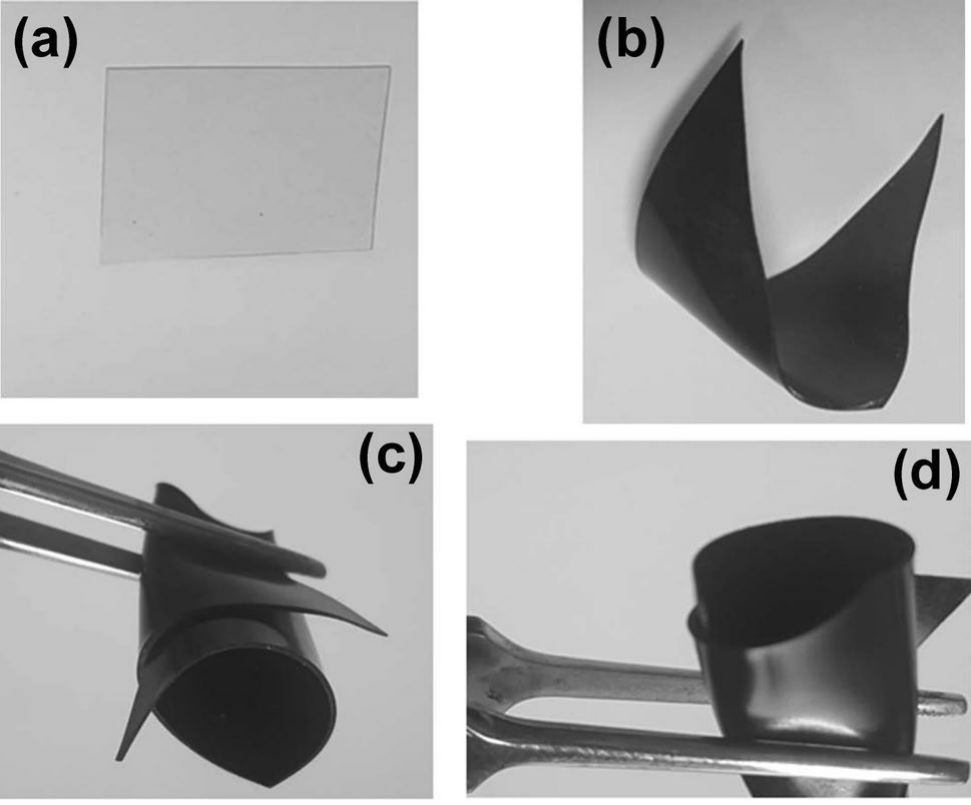
The photography images of neat EPR (**a**) and GNS/EPR_1–5_ (**b**–**d**)

Increasing the GNS-content to GNS/EPR_3_ is accompanied by an emergence of small reflection at 2*θ *= 26.57°, attributable to the main XRD-peak for GNS (Fig. 1e). Further increase in the GNS wt% till 20% leads to a continuous shift of that reflection to 2*θ *= 24.81° (Fig. 1g), which indicates a larger interlayer spacing (0.3574 nm) than that of the bare GNS; meanwhile, the patterns for the various composites still showing the features characterizing the EPR. The detection of these peaks indicates that the structure of neither graphene nor the epoxy resins was not destroyed during the composite-preparation process. Concurrently, Yu et al. [51] reported a shift in the XRD main peak of GO from 10.0° to 9.6° upon anchoring Al_2_O_3_ on GO sheets. They have correlated this shift to the formation of the disordered and loosened sheet-like structure of GO [51]. Figure 2 showed the photography images of neat EPR and GNS/EPR_1–5_, which indicated that we have successfully fabricated flexible sheets with a very simple method that allowed different applications for these nanocomposites sheets with different amounts of GNS.

**Fig. 3:**
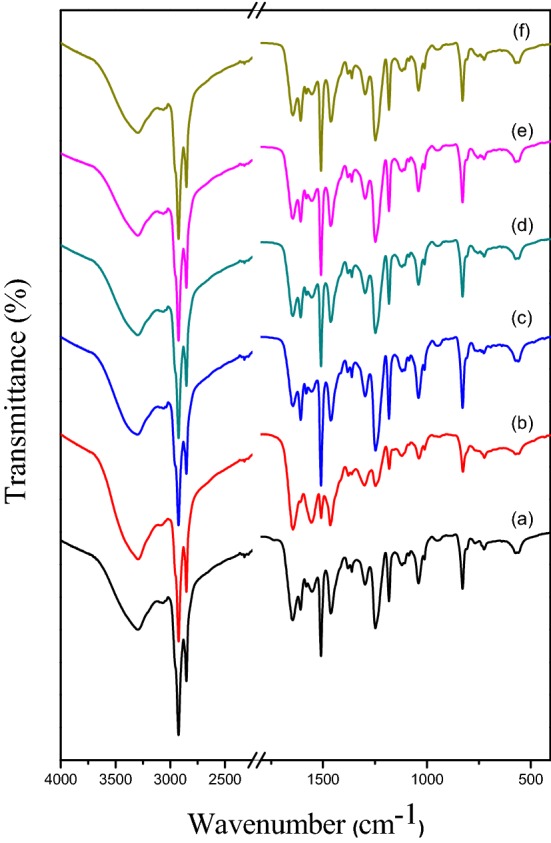
FT-IR spectra of neat EPR (**a**), GNS/EPR_1_ (**b**), GNS/EPR_2_ (**c**), GNS/EPR_3_ (**d**), GNS/EPR_4_ (**e**) and GNS/EPR_5_ (**f**)

FT-IR spectra for the neat EPR and its GNS/EPR_1–5_graphene-containing nanocomposites are measured using ATR smart pat over the range of 4000 to 400 cm^−1^. A visible proof for the expected bonding interaction between neat EPR and its GNS/EPR_1–5_ graphene-containing nanocomposites is given in Fig. 3. The neat EPR spectrum shows all characteristic absorption peaks as reported in our previous work [32, 43, 44] and as reported in the open literature [52, 53]. Peaks at 3050–3015 cm^−1^, 3045 cm^−1^, and 2965 cm^−1^ are attributed to the valent CH vibrations of the epoxy ring, the stretching CH vibration of the aromatic ring, and the stretching vibration of the –CH_2_ functional group respectively. Furthermore, other peaks at 1245 cm^−1^ and in the range 930–815 cm^−1^ for the valent CO vibrations of the epoxy ring, and the bending CH vibrations of the epoxy ring respectively. Moreover, Fig. 3 also asserts such type of interaction where it shows the significant characteristic peaks of GNS in the spectra of GNS/EPR_1–5_ nanocomposites [54–57]. Besides that, EPR characteristic peaks are also mentioned in the spectra of these products. FT-IR spectra of GNS/EPR_1–5_ in Fig. 3 also display that, a major decrease in the intensity of neat EPR characteristic peaks is obviously observed and vice versa for GNS immersed fillers.

**Fig. 4:**
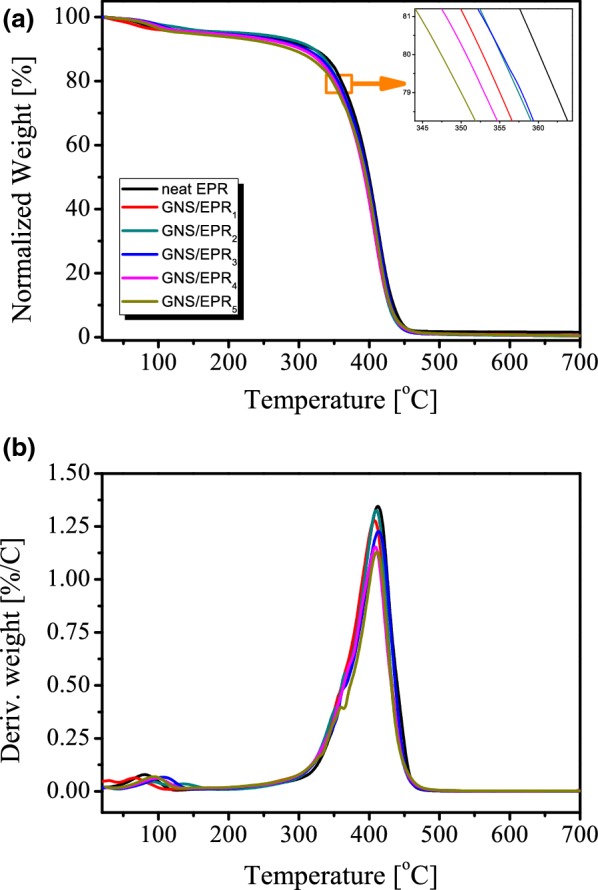
TGA (**a**) and DTG (**b**) thermoanalytical curves of neat EPR and its GNS/EPR_1–5_ graphene-containing nanocomposites

TGA was used to investigate the thermal stability of the neat EPR and its various GNS/EPR_1–5_nanocomposites over the temperature range of 21–700 °C. Figure 4a shows plots of the normalized weight loss (NWL) percentages versus temperature. The NWL is defined as [(w_T_/w_G_)/(w_init_/w_G_)] × 100, where w_T_ is the weight at temperature T, w_G_ is the graphene weight, and w_init_ is the sample initial weight. All the obtained thermograms show two weight loss steps. The first one extends from ambient to 180 °C and amounts to 4.5–5.8%, which could be attributed to the evolution of impurity traces apart from the cured epoxy resin together with the elimination of water molecules [43, 44]. The second weight-loss step, which is the main step that is characterized by a steep weight change, takes place at the temperature range of 250–500 °C. This step could be assigned to the chain scission together with resin decomposition yielding lower molecular weight products [43, 44]. The obtained TGA curves of the fabricated GNS/EPR_1–5_ nanocomposites are very similar to that of neat EPR. Table 2 lists the T_2_, T_20_ (temperatures corresponds to 2%, 20%, and weight loss, respectively) values of neat EPR and its graphene-based nanocomposites. Inspection of these values reveals that the presence of GNS content higher than 3% retards the early weight loss step of the neat epoxy. On the other hand, the T_20_ values show a 5–13 °C shift toward lower values because of GNS incorporation.

**Table 2:** Thermoanalytical data of neat EPR and its GNS/EPR_1–5_ graphene-containing nanocomposites under nitrogen flow

Sample	T_2_ [°C]	T_20_ [°C]	T_max_ [°C]
neat EPR	74	360	412
GNS/EPR_1_	60	353	407
GNS/EPR_2_	95	355	410
GNS/EPR_3_	93	355	409
GNS/EPR_4_	87	350	409
GNS/EPR_5_	81	347	410

Inconsistent thermal-stability trends have been reported in the literature for the incorporation of epoxy with graphene-based materials [46, 48, 49, 58–60]. For instance, Wang et al. [58] reported that, in comparison with the neat epoxy, the SnO_2_–graphene/epoxy and Co_3_O_4_–graphene/epoxy composites exhibited a 37 and 27 °C increment in the onset decomposition temperature, respectively. This increased thermal stability was ascribed to the presence of a metal oxide-G synergic effect. Liu et al. [46] investigated the thermal degradation behavior of the epoxy composites with slightly oxidized graphene (graphenit-ox) and Cu-doped graphene (graphenit-Cu). Their results revealed that the incorporation of graphenit-ox (1–3 wt%) into epoxy resin led to slight thermal stability enhancement. On the other hand, the graphenit-Cu exhibits a catalytic effect, i.e., it decreases the epoxy decomposition temperature. Wan et al. [49] showed that the GO/epoxy composites formation increases the decomposition temperature (T_d_) of epoxy resin by 12–16 °C. Moreover, the GO loading (0.1–0.5 wt%) did not appreciably affect that temperature [49]. On the other hand, an increase in the T_d_ value by around 30 °C was obtained on functionalizing the GO/epoxy composites with diglycidyl ether of bisphenol-A [49]. The recent work of Gong et al. [48] indicated that, in comparison with neat epoxy, the decomposition temperature of epoxy/RGO (0.5 wt% loading) is slightly increased by 12 °C. Replacing RGO with powdered rubber in a 3% and 6% loading led to ~ 54 °C and ~ 104 °C decrease in the decomposition temperature [48]. An and Jeong [59] reported a slight decrease in the decomposition temperature of G/epoxy composites (0.3-7 wt% loading) compared to the bare epoxy resin. Zhang et al. [60] reported a ~ 1 °C increase of the onset decomposition temperature (T_onset_) of G/epoxy composite (loading 1.0 wt%) compared to the bare epoxy. Further increase in the G loading to 5 wt% in the form GNS/EPR_2_ led to a ~ 5.5 °C decrease in the T_onset_ value [60]. Moreover, the incorporation of Fe@Fe_2_O_3_ to the G/epoxy composites in loadings of 1, 3 and wt% was accompanied by 13, 21 and 19 °C decrease in the T_onset_ value [60]. Table 2 also lists the T_max_ (maximum decomposition temperature) values of neat EPR and its GNS/EPR_1–5_ graphene-containing nanocomposites [32, 61–63]. T_max_ values were calculated from the DTG thermoanalytical curves (see Fig. 4b). T_max_ values are nearly similar for EPR and its GNS/EPR_1–5_ nanocomposites which ranged from 407 to 412 °C. This is due to the similar decomposition pattern for all materials (two main degradation steps) as previously discussed. The pure EPR shows somewhat the highest T_max_ value while GNS/EPR_1_ nanocomposite shows the lowest value. In conclusion, our results clearly indicate that the presence of GNS slightly decreases the thermal stability of neat EPR. Moreover, a dependence of the T_20_ value on the GNS content in the composite form is evident. The T_20_ decreases on increasing the GNS to 5–10 wt%; higher content leads to a slight increase in the T_20_ value.

**Fig. 5:**
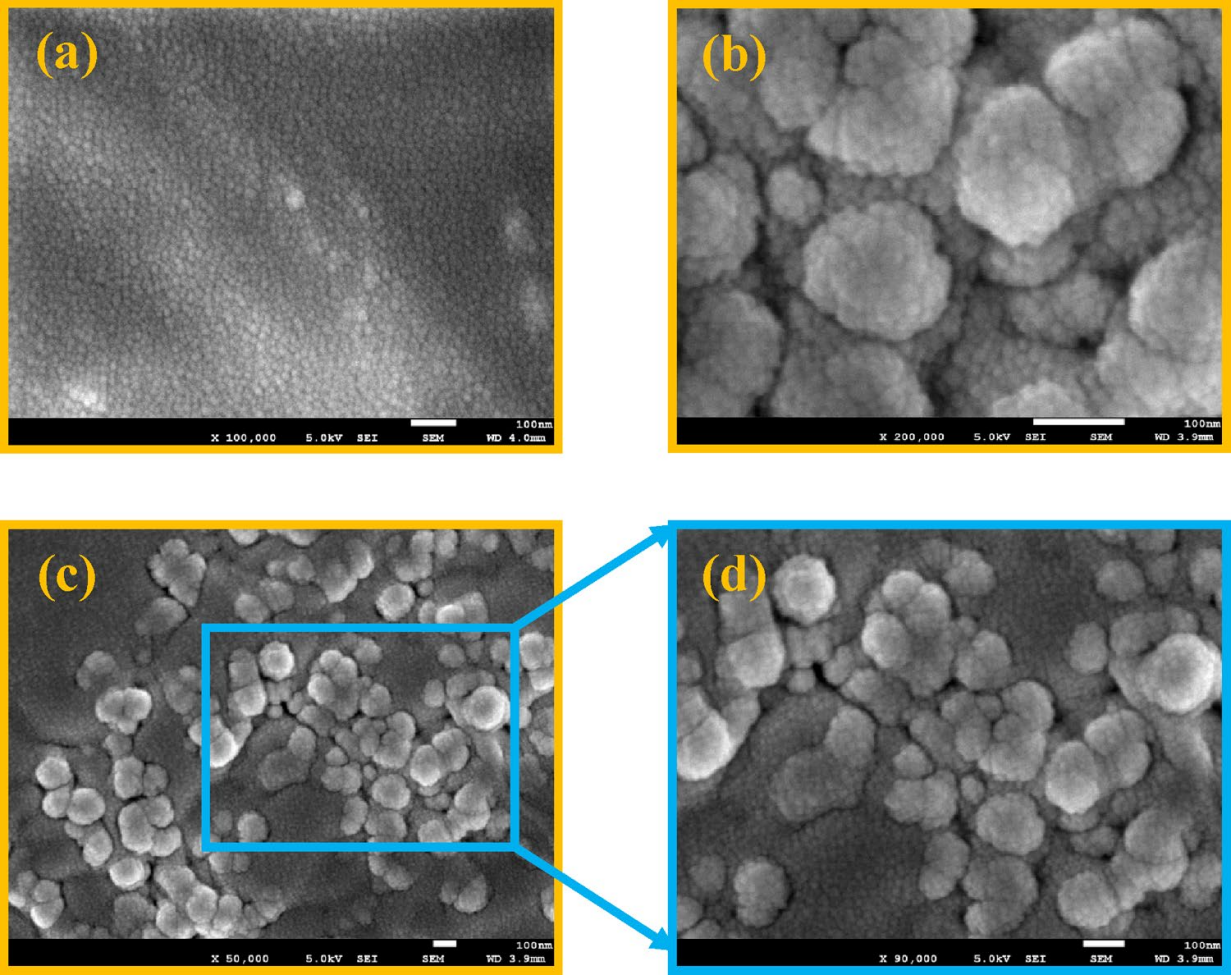
FE-SEM images for GNS/EPR_2_**a** x = 100,000, **b** x = 200,000 and for GNS/EPR_5_, **c** x = 50,000, **d** x = 90,000

Field emission scanning electron microscopy (FE-SEM) is applied to study the morphological surface changes in the fabricated GNS/EPR_1–5_ graphene-containing nanocomposites. The FE-SEM images show a visible morphological directory for the formation of our designed materials. Fe-SEM micrographs are picked up using a Penta Z Z-50P Camera with Ilford film at an accelerating voltage of 5 kV and a nearly 4 mm work distance carbon film using a low dose technique [64]. The FE-SEM micrographs are illustrated in Fig. 5a–d for GNS/EPR_2_and GNS/EPR_5_as the second lowest and the highest loading of GNS. It is clearly noticed from the images that, the neat EPR surface shows a smooth surface under higher magnification x = 100,000; while in very high magnification (x = 200,000), the surface shows tiny spherical particles. Whereas, the dispersion states of the GNS in the neat EPR are easily demonstrated in the FE-SEM images of GNS/EPR_2_ and GNS/EPR_5_ as illustrated in Fig. 5a, b. Such FE-SEM micrographs display a valuable surface modification upon GNS inundation inside the EPR polymer matrix. Figure 5 also shows GNS is uniformly dispersed throughout the EPR polymer matrix in the given magnifications. This exposes excellent miscibility between EPR (organic) and GNS (inorganic) parts in the nanocomposites production. A greater sense of compatibility, distribution pattern of the GNS is also specified by these micrographs. Meanwhile, good cohesion between this filler particle and the polymer matrix is also observed. Whoever, the images also show slightly aggregated particles that are attributed to the relatively higher loading of GNS that are found in the composition of GNS/EPR_5_ as given in Fig. 5c, d.

### Characterizations of different Au NPs/GNS/EPR nanocomposites sheets

**Fig. 6:**
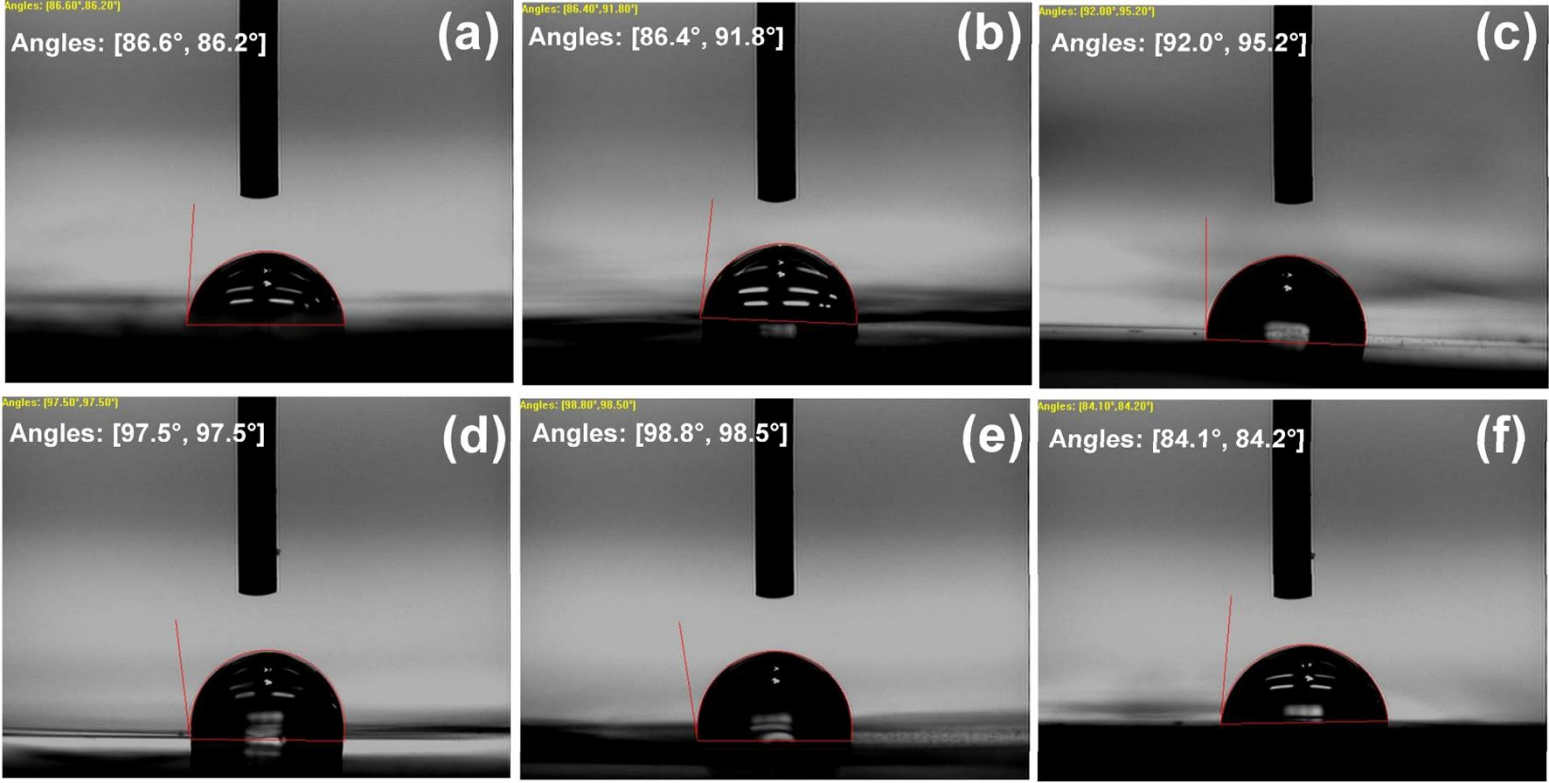
Images contact angles of water with neat EPR (**a**), GNS/EPR_1_ (**b**), GNS/EPR_2_ (**c**), GNS/EPR_4_ (**d**), GNS/EPR_5_ (**e**) and Au NPs/GNS/EPR_5_ (**f**)

The wettability of developed sensor is one of the important futures in order to allow the immunization of the target species; thus the effect of the amount of the loading GNS on the contact angle between water and the GNS/EPR in addition to the contact angle of Au NPs/GNS/EPR were studied. Figure 6 showed the images of the water contact angles with the different modified GNS/EPR substrate in comparison with the bare resin substrate. The values of the contact angles were summarized in Table 3, which indicated that the contact angle value was increased with increasing the amount of loading of GNS and hence the hydrophobic characteristic will increase that restricted the immobilization of the target species from the aqueous medium. On the other hand, the deposition of Au NPs results in decreasing the contact angle in comparing with the all GNS/EPR_1–5_ substrates as well as the bare resin substrate.

**Table 3:** The contact angle data of neat EPR, GNS/EPR_1–5_ nanocomposites and its Au NPs/GNS/EPR with water

Sample	Right contact angle (°)	Left contact angle (°)	Average contact angle (°)
Pure EPR	86.6	86.2	86.4
GNS/EPR_1_	86.4	91.8	89.1
GNS/EPR_3_	92.0	95.2	93.6
GNS/EPR_4_	97.5	97.5	97.5
GNS/EPR_5_	98.8	98.5	98.65
Au NPs/GNS/EPR	84.1	84.2	84.15

### The enhancement factor of the different modified sheets

**Fig. 7:**
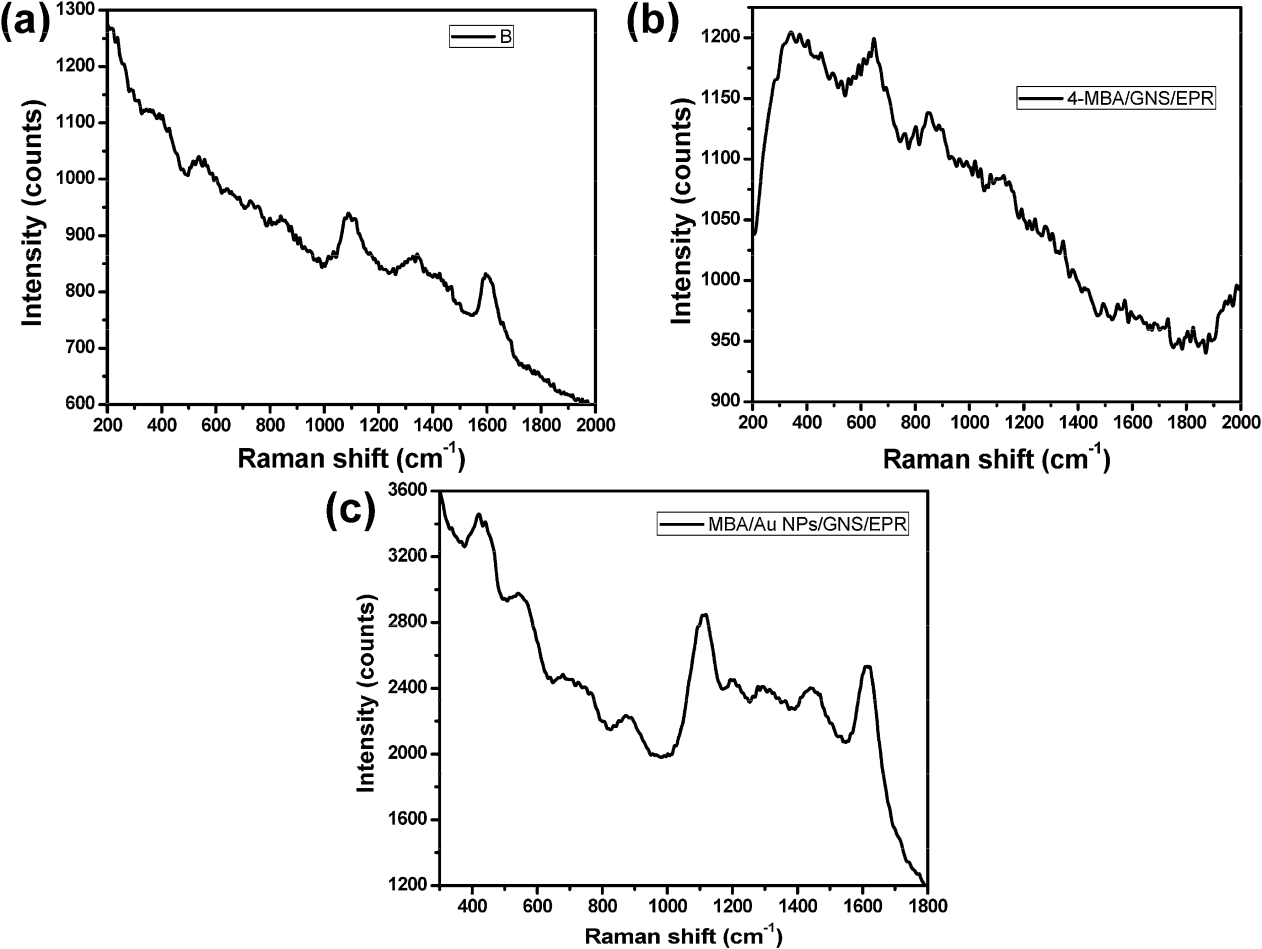
Raman spectra of 2 mM of 4-MBA with neat EPR (**a**), GNS/EPR_5_ (**b**), and Au NPs/GNS/EPR_5_ (**c**)

In order to calculate the enhancement factor (EF), a solution of 2 mM of 4-mercaptobenzoic acid (4-MBA) in ethanol was immobilized onto the different modified substrates (pure resin, GNS/EPR_5_ and Au NPs/GNS/EPR_1–5_) and then the Raman spectra were recorded by using the same laser power and accumulations time. The EF was calculated based on the following equation EF = (SERS intensity x effective SERS analytes)/(Raman intensity x effective Raman analytes). Figure 7a showed the Raman spectrum of 2 mM of 4-MBA immobilized onto the pure resin, which showed a very poor Raman signal. While the GERS spectrum of 2 mM of 4-MBA immobilized on GNS/EPR_5_ substrate showed a little enhancement of the Raman signals (Fig. 7b). On the other hand, immobilization of 2 mM of 4-MBA onto Au NPs/GNS/EPR_5_ modified substrate showed the highest enhancement that related to the presence of Au NPs (Fig. 7c), which serves as hotspots for improving the intensity of the Raman signal and also as nanoplatforms for immobilization of 4-MBA based on the direct interaction with thiol groups. The corresponding EF was found to be 6.2 × 10^6^.

### Monitoring of different concentrations of dopamine neurotransmitter

**Fig. 8:**
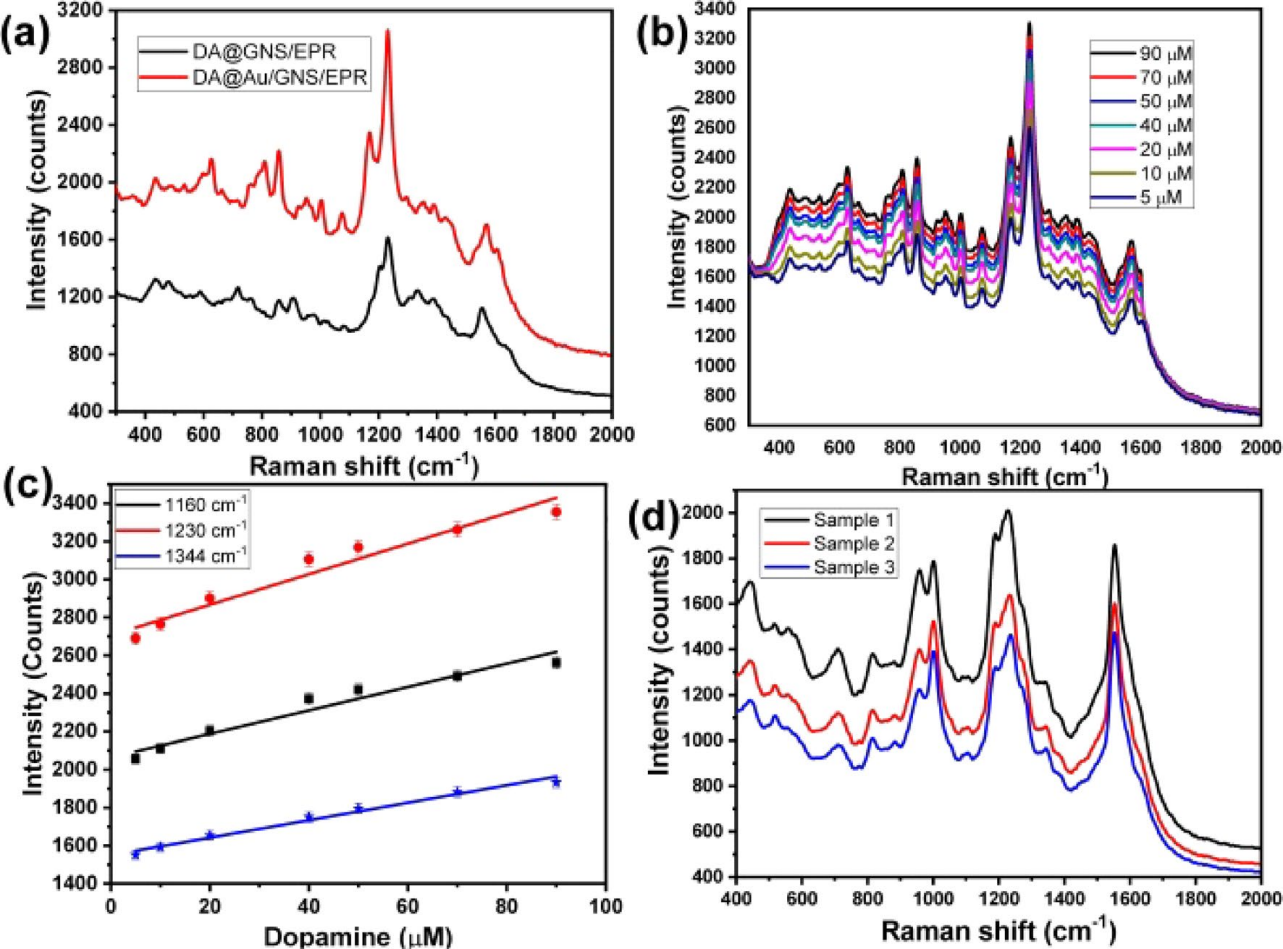
Raman spectra of dopamine solution in PBS buffer by using GNS/EPR_5_ and Au NPs/GNS/EPR_5_ (**a**), G-SERS spectra of different concentrations of dopamine dissolved in PBS buffer by using Au NPs/GNS/EPR_5_ (**b**), the relationship between the intensities of SERS signals (857, 1074 and 1344 cm^−1^) and the corresponding concentration of dopamine (**c**), and G-SERS spectra of different concentrations of dopamine dissolved in human serum by using Au NPs/GNS/EPR_5_ (**d**)

The Raman spectrum of Au NPs/GNS/EPR (Fig. 7c) was as recorded a blank spectrum and subtract before measure the Raman spectra of dopamine. Fifty microliters of dopamine solutions with different concentrations were self-assembled on GNS/EPR as well as Au NPs/GNS/EPR and then the SERS spectra were recorded five times and the average data were represented in Fig. 8a, displayed several Raman bands as following 597 cm^−1^ (in-plane ring deformation), 627 cm^−1^ (CH wagging; aliphatic chain C–C vibrations), shoulder Raman band 757 cm^−1^, 805 cm^−1^ (CH out-of-plane; ring deformation), 927 cm^−1^ (NH twisting), 1160 cm^−1^ (OH rocking; CH aromatic rocking; weak ring breathing; CH wagging), 1230 cm^−1^ (CO stretching), 1430 cm^−1^ (CH scissoring) and 1620 cm^−1^ (NH_2_ scissoring), which are in good concordant with the previous reported spectra data [40]. Furthermore, it was easily note that the uses of Au NPs/GNS/EPR as SERS-active substrate results in highly enhancement compared with the GNS/EPR. Figure 8b displays the SERS spectra of different concentrations of dopamine that illustrated an increase in the Raman intensities with increasing the dopamine concentration. Figure 8c represented the relationship between the concentration of dopamine within a range from 5 µM to 90 µM and the intensity of the GSERS signals at 1160, 1230 and 1344 cm^−1^, which displayed a linear relationship with an R^2^ of about 0.954, 0.951 and 0.983, respectively. Thus the intensity of peak 1344 cm^−1^ showed the highest successive changes depending on the change in dopamine concentration. The limit of detection (LOD) of the dopamine was calculated as following, (LOD = 3.3*(STEYX/Slope of calibration curve)), and it was found equal to 3.3 µM.

### Monitoring of dopamine in human serum sample as a model of real samples

The capability to use this sensor to detect dopamine neurotransmitters in a real sample was studied based on monitoring low concentrations of dopamine in human serum as a represented real sample. Figure 8d showed the Raman spectra of three different concentrations of dopamine soluble in human serum solution. The Raman spectra displayed a set of Raman bands similar to the Raman spectrum of dopamine dissolved in PBS buffer with a little difference in the strength of some bands, which established the capability of the Au NPs/GNS/EPR to apply for monitoring dopamine in complicated media such as human serum sample without any interference-effect.

## Conclusions

Here we have successfully prepared fillable sheets congaing different amounts of GNS based one-step process in which the epoxy resin (EPR) was fabricated by ultrasonic-assisted and the graphene nanosheets were added while the epoxy resin was formed. The obtained GNS/EPR sheet was decorated with Au NPs to enhance the Raman sensitivity and to allow the immobilization of the target species. This flexible sheet was used for direct monitoring of different concentrations of dopamine neurotransmitters in human serum, thus this flexible sensor can be applied to other biomedical wearable sensor applications.

## Data Availability

Not applicable.

## Abbreviations

SERS: Surface-enhanced Raman spectroscopy
EPR: Epoxy resin
GNS: Graphene nanosheets
GNS/EPR: Graphene nanosheets/epoxy resin
Au NPs/GNS/EPR: Gold nanoparticles/graphene nanosheets/epoxy resin
DA: Dopamine
HAuCl_4_·4H_2_O: Chloroauric acid tetrahydrate
NaBH_4_: Sodium borohydride
CCD: Charge-coupled device
FT-IR: Fourier-transform infrared spectroscopy
XRD: X-ray diffraction
TGA: Thermogravimetric analysis
RGO: Reduced graphene oxide
PR: Powdered rubber
DGEBA-f-GO: Diglycidyl ether of bisphenol-A functionalized graphene oxide
Al_2_O_3_: Aluminum oxide
NWL: Normalized weight loss
T_max_: The maximum decomposition temperature
T_onset_: The onset decomposition temperature
FE-SEM: Field emission scanning electron microscopy
EF: Enhancement factor
4-MBA: 4-mercaptobenzoic acid
LOD: The limit of detection
